# Factors associated with participation in a proton therapy clinical trial: a cross-sectional survey of Danish patients with head and neck cancer

**DOI:** 10.2340/1651-226X.2025.43912

**Published:** 2025-07-10

**Authors:** Anne Wilhøft Kristensen, Kenneth Jensen, Annesofie L. Jensen, Susanne O. Dalton, Jesper G. Eriksen, Jeppe Friborg, Cai Grau

**Affiliations:** a Danish Centre for Particle Therapy, Aarhus University Hospital, Aarhus, Denmark; b Steno Diabetes Centre, Aarhus University Hospital, Aarhus, Denmark; c Department of Clinical Medicine, Health, Aarhus University, Aarhus, Denmark; d Cancer Survivorship, Danish Cancer Institute, Copenhagen, Denmark; e Department of Clinical Oncology & Palliative Care, Zealand University Hospital, Næstved, Denmark; f Institute for Clinical Medicine, Faculty of Health, Copenhagen University, Copenhagen, Denmark; g Experimental Clinical Oncology, Aarhus University Hospital, Aarhus, Denmark; h Department of Clinical Oncology, Rigshospitalet, Copenhagen, Denmark; i Danish Centre for Particle Therapy, Aarhus University Hospital, Aarhus, Denmark

**Keywords:** neoplasms, head and neck, radiotherapy, proton beam, clinical trials as topic, patient participation, health literacy, surveys and questionnaires, socioeconomic factors

## Abstract

**Background and purpose:**

Participation in proton therapy (PT) trials may be affected by structural, clinical, and individual factors, potentially excluding certain patient groups. Such disparities can lead to unequal access to potential research benefits and may limit the generalisability of trial findings. This study aimed to identify factors associated with participation in a Danish randomised controlled trial (RCT) comparing proton versus photon radiotherapy for head and neck cancer.

**Patients and methods:**

This national cross-sectional study invited patients with pharyngeal and laryngeal cancer, referred for curative-intent radiotherapy at seven Danish radiotherapy clinics between 2022 and 2025, to complete a survey. Respondents were categorised based on enrollment status in a national RCT comparing proton versus photon radiotherapy. Clinical, demographic, psychosocial, and lifestyle data were collected and linked to clinical registry data. Multiple logistic regression was used to assess exposure variables associated with trial participation.

**Results:**

Of 304 respondents, 120 (39%) were enrolled in the RCT. Female gender, older age, greater geographical distance to the PT centre, mobility limitations, lower self-rated health status, and lower ability to actively engage with healthcare providers (Health Literacy Questionnaire scale 6) were significantly associated with lower odds of participation. No significant associations were observed for income, education, marital status, or anxiety.

**Interpretation:**

The findings indicate that demographic, geographical, functional, and communication-related factors may limit participation in PT trials. This highlights the need for interventions that enhance the delivery of trial information, strengthen communication between patients and healthcare professionals, and support informed clinical trial decision-making.

## Introduction

Radiotherapy with protons holds great promise in cancer treatment and has been a growing focus of cancer research in recent years. However, access to proton therapy (PT) trials may be skewed by geographical, socioeconomic, and psychosocial factors.

Patients with smoking-induced head and neck cancer generally have lower levels of education and income and are more likely to live alone compared to the general population [1–4]. Such socioeconomic factors have been shown to affect access to treatment opportunities and are associated with lower 5-year survival rates [3–5].

Radiotherapy is the primary treatment for patients with pharyngeal and laryngeal cancers [6]. While photon-based radiotherapy is effective, it significantly impacts the quality of life due to long-term treatment-induced toxicities [7]. PT reduces radiation exposure to healthy tissues, potentially reducing long-term treatment-related toxicities [8]. Denmark has one centralised PT facility. Like other hospital treatments, it is publicly funded and provided at no cost to the patient. In recent years, clinical trials have been initiated for various cancer sites to evaluate the clinical potential of PT before routine implementation. For Danish patients with pharyngeal and laryngeal cancer, participation in an ongoing clinical trial is currently the only pathway to accessing PT [9].

Clinical trial barriers such as geographical distance, socioeconomic position, comorbidity, and reduced quality of life have been reported in prior studies [10–17]. Health literacy may further contribute to these disparities, as patients with limited health literacy are less frequently invited to participate despite their willingness [18, 19]. Explanations may include difficulties understanding trial information or limited ability to communicate effectively with healthcare professionals. Additionally, anxiety or distress may impair patients’ ability to cope with trial participation, potentially affecting their decision to enrol [20, 21].

Such disparities in trial participation not only raise ethical concerns about unequal access to potential research benefits but may also limit the external validity of trial findings [22].

This population-based study aimed to identify factors associated with PT clinical trial participation among Danish patients with pharyngeal and laryngeal cancer.

## Method

### Study design

This study was a cross-sectional survey that collected prospective data from newly diagnosed patients at seven Danish radiotherapy clinics.

### Context

The Danish Head and Neck Cancer Study Group (DAHANCA) initiated a randomised controlled trial (RCT), named DAHANCA 35, in 2020, serving as this study’s clinical case.

DAHANCA 35 compares radiotherapy with photons and protons for treating pharyngeal and laryngeal cancer, with xerostomia and dysphagia as primary endpoints. Patient eligibility for DAHANCA 35 was determined based on a comparative photon and proton treatment plan. In case of a predicted theoretical benefit of PT, the patient is eligible for inclusion and randomisation [9, 23]. The trial was designed to include a broader patient population beyond human papillomavirus-positive oropharyngeal cancer, increasing the need for representative recruitment.

Patients were randomised in DAHANCA 35 at six Danish radiotherapy clinics located at varying distances from the PT facility: Hospital 1 (318 km), Hospital 2 (307 km), Hospital 3 (244 km), Hospital 4 (145 km), Hospital 5 (0 km; co-located with Danish Centre for Particle Therapy), and Hospital 6 (115 km).

### Survey participants

Eligible survey participants comprised Danish-speaking adult patients (≥ 18 years) diagnosed with pharyngeal (nasopharyngeal, oropharyngeal, hypopharyngeal) or laryngeal cancer (excluding stage I/II glottic larynx) and referred for curative-intent radiotherapy. These patients were eligible for the survey regardless of their inclusion in DAHANCA 35.

### Survey distribution

To promote consistent survey distribution across centres, the first author (AWK) trained clinic staff in survey purpose, eligibility, and procedures, and maintained quarterly contact throughout data collection. Eligible survey participants were approached during the first 2 weeks of radiotherapy and received the questionnaire with a prepaid return envelope.

### Data collection methods

Participants completed the questionnaire on a paper-based version or online via REDCap [24, 25]. Data from the paper-completed questionnaires were entered into REDCap by AWK. Patient identity and eligibility were verified by linking questionnaire responses to the DAHANCA database using the Danish social security number as an identifier. Clinical data were extracted from the DAHANCA database [26].

### Demographic and exposure variables

Questionnaire data included sociodemographic, residential, and lifestyle factors such as age, sex, marital status, education, employment status, place of residence (region and postal code), local hospital, and alcohol and smoking habits.

Age was analysed as a continuous variable, expressed as the mean, and as a categorical variable using the following groups: < 55 years, 55–70 years, and > 70 years. Cohabitation status was dichotomised as living with a partner or alone.

Educational level was categorised based on the International Standard Classification of Education (ISCED), grouping ISCED levels 1–2 as short education (mandatory school, 7–9 years), levels 3–4 as medium education (youth or vocational education, 8–13 years), and levels 5–7 as long education (higher education, > 13 years) [27].

Employment was categorised into three groups: (1) active in the workforce, including individuals under education, on sick leave, or temporarily unemployed; (2) retired due to age; and (3) reduced work capacity, including individuals in supported employment or receiving a disability pension.

Yearly income was dichotomised at 200,000 Danish kroner (approximately 30,000 US Dollars) to distinguish individuals following the definition of low income. According to Denmark’s official definition of low income, a household-equivalised disposable income below 50% of the national median income is considered low-income [28].

Anxiety was measured using the Danish validated version of GAD-7 (Generalised Anxiety Disorder-7), which is a 7-item scale assessing anxiety severity. Scores range from 0 to 21, with higher scores indicating greater anxiety levels [29]. Anxiety was presented as a continuous variable, expressed as the median (interquartile range), and as a categorical variable with sum scores categorised into four thresholds: 0–4 (minimal anxiety), 5–9 (mild anxiety), 10–14 (moderate anxiety), and 15–21 (severe anxiety) [29].

Health literacy was assessed using two subscales of the Health Literacy Questionnaire (HLQ), which is validated in a Danish population. The two subscales used were 6: *‘Ability to actively engage with healthcare providers’* and 9: *‘Understand health information well enough to know what to do’*. Scores range from 1 to 5, with higher scores reflecting better health literacy [30, 31]. Since no validated cutoff has been established, the continuous mean HLQ variable is used in the regression analysis [30].

Health-related quality of life (HRQoL) was evaluated using the Danish validated version of the EuroQoL (EQ-5D-5L) questionnaire, which includes a 0–100 visual analogue scale (VAS) to assess overall health status for the day [32, 33]. The five dimensions in EQ-5D-5L were dichotomised as ‘no problems’ or ‘any problems’, while the VAS score was presented as a continuous variable.

Smoking habits were categorised as current, former, and never. Alcohol consumption was dichotomised as below or above seven units per week for women and 14 units per week for men, reflecting the Danish Health Authority’s recommendations at the time the questionnaire was developed.

Distance to the PT facility was assessed in three ways. First, the five Danish regions were included as a categorical variable and subsequently dichotomised into Eastern and Western Denmark, separated by the Great Belt; the PT facility is located in Western Denmark. Second, the distance from the patient’s place of residence (postal code) to the PT centre was included as a continuous variable. Third, distance was categorised as short (< 50 km), medium (50–150 km), or long (> 150 km), reflecting increasing travel burden. Distances greater than 150 km are often assumed to necessitate accommodation [13]. Finally, a fourth measure was included to capture the relative travel burden: the individual difference in travel distance (in kilometres) between the patient’s home and the nearest photon-based Radiotherapy (RT) facility versus the PT centre.

Clinical data included tumour site (nasopharyngeal, oropharyngeal, hypopharyngeal, or laryngeal cancer), disease stage (I–IV), p16 status for oropharyngeal cancer, and co-morbidity [26]. Comorbidity was assessed using the Charlson Comorbidity Index and dichotomised as no comorbidity or ≥ 1 comorbidity [34].

### Outcome

The outcome variable was participation in DAHANCA 35. Patients were categorised into two groups: (1) participants and (2) non-participants in DAHANCA 35. Participants were the patients randomised, while non-participants included those who were not informed about the trial, ineligible, or declined participation.

### Sample size

The sample size was determined based on a previous survey comparing participants and non-participants in a cancer clinical trial [17]. In that study, 12% of participants and 25% of non-participants had a short education. To detect a similar difference in this study, with a power of 0.8 and an alpha of 0.05, responses from a minimum of 130 trial participants and 200 non-participants were required.

### Statistical analysis

Demographic, clinical and health-related variables were analysed using descriptive statistics. Categorical variables are presented as frequencies and percentages, and normally distributed continuous variables as means with standard deviations (SD). Comparisons between participants and non-participants were performed using chi-squared tests for categorical variables and independent samples *t*-tests for normally distributed continuous variables. To illustrate participation patterns in the cross-sectional survey, participant/non-participant ratios were calculated per hospital.

For each exposure variable, univariable logistic regression was used to assess its association with trial participation. Subsequently, separate multivariable logistic regression models were performed for each exposure, adjusting for age and sex to account for potential confounding. This approach allowed for the evaluation of individual associations without overfitting. Results are reported as odds ratios (OR) with 95% confidence intervals (CI).

## Results

**Figure 1:**
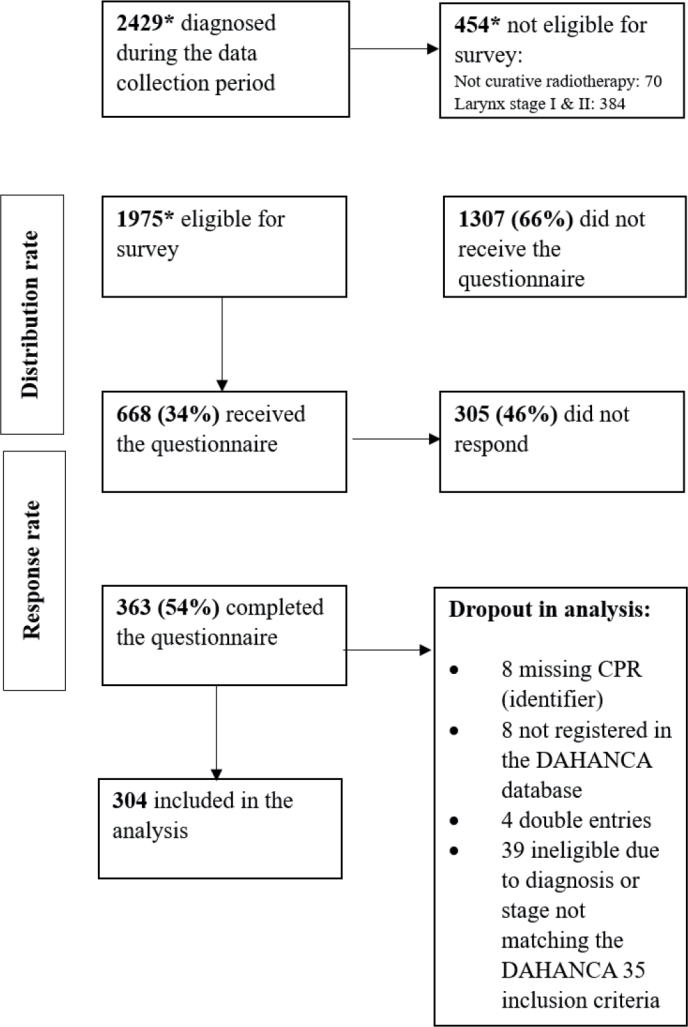
Distribution and response rate in the survey. DAHANCA: Danish Head and Neck Cancer Study Group. *Data retrieved from the DAHANCA database.

Between March 2022 and April 2025, a total of 2,429 patients were diagnosed with pharyngeal (nasopharyngeal, oropharyngeal, hypopharyngeal) or laryngeal cancer in Denmark. Of these, 1,975 patients were eligible for the survey, excluding those with non-curative treatment intent or stage I/II glottic larynx cancer. 668 (34%) received the questionnaire. Of those, 363 patients completed it, yielding a response rate of 54%, with centre-specific response rates ranging from 37% to 71%. Following data cleaning, 304 responses were included in the final analysis. Survey distribution, response rates, and exclusions before analysis are summarised in Figure 1.

Of the 304 respondents included in the analysis, 120 were randomised in the DAHANCA 35 trial and classified as ‘participants’ in the survey. The remaining 184 were classified as ‘non-participants’. Among these, 41 respondents indicated not to have been informed about DAHANCA 35, with the following distribution across centres (non-informed out of total respondents): Hospital 1 (5 of 73, 7%), Hospital 2 (7 of 36, 19%), Hospital 3 (13 of 60, 22%), Hospital 4 (8 of 50, 16%), Hospital 5 (3 of 38, 8%), and Hospital 6 (4 of 47, 9%). The final sample thus comprised 120 participants (39%) and 184 non-participants (61%) (Table 1).

**Table 1:** Survey respondents, grouped as participants and non-participants, based on DAHANCA 35 participation status.

DAHANCA 35 status among the survey respondents		Survey groups	
Not informed about DAHANCA 35	41	184 (61%)	Non-participants
Not enrolled in DAHANCA 35	143		
Randomised to DAHANCA 35	120	120 (39%)	Participants

DAHANCA: Danish Head and Neck Cancer Study Group.

Table 2 presents the demographic, clinical, and lifestyle characteristics of participants and non-participants in the DAHANCA 35 trial (Table 2).

**Table 2:** Characteristics of participants and non-participants in the RCT.

Variables	Participants, *N* (%)	Non-participants, *N* (%)	*P*-value
Gender (*N* = 304)			0.04
Female	20 (23%)	49 (27%)	
Male	100 (77%)	135 (73%)	
Age (years), mean (SD)	63 (8.26)	66 (9.45)	0.01
Age (*N* = 304)			0.01
< 55	21 (18%)	23 (12%)	
55–70	70 (58%)	88 (48%)	
> 70	29 (24%)	73 (40%)	
Survey recruitment centre				< 0.001
*N* (%, ratio*)				
Hospital 1	23 (19%, ratio 0.45)	51 (28%)	
Hospital 2	12 (10%, ratio 0.60)	20 (11%)	
Hospital 3	15 (12%, ratio 0.47)	32 (18%)	
Hospital 4	26 (22%, ratio, 0.81)	32 (18%)	
Hospital 5	36 (30%, ratio 1.89)	19 (10%)	
Hospital 6	8 (7%, ratio 0.29)	28 (15%)
Distance to RT			0.3 0.001
(median km, range)			
Photons	37 (11–66)	32 (8–59)	
Protons	169 (86–300)	271 (140–305)	
Diagnosis (*N* = 304)			0.3
Laryngeal cancer	3 (3%)	11 (6%)	
Oropharyngeal cancer	109 (91%)	149 (81%)	
Nasopharyngeal cancer	3 (3%)	1 (< 1%)	
Hypo-pharyngeal cancer	5 (3%)	23 (12%)	
Tumour stage (*N* = 302)			0.2
T1–T2	84 (70%)	114 (63%)	
T3	22 (18%)	33 (18%	
T4	14 (12%)	35 (19%)	
Nodal stage (*N* = 302)			0.08
N0	8 (7%)	29 (16%)	
N1	73 (61%)	96 (53%)	
N2	33 (27%)	44 (24%)	
N3	6 (5%)	13 (7%)	
Stage (*N* = 300)			0.7
St 1	52 (43%)	74 (41%)	
St 2	25 (21%)	32 (18%)	
St 3	21 (18%)	34 (19%)	
St 4	21 (18%)	41 (22%)	
HPV status – oropharyngeal (*N* = 254)				0.09
P-16-positive	95 (87%)	118 (81 %)		
P-16 negative	14 (13%)	21 (15%)	
P-16 status unknown	0 (0%)	6 (4%)
Co-morbidity (*N* = 304)			0.2
None	83 (69%)	113 (61%)	
≥ 1 co-morbidity	37 (31%)	71 (39%)	
Health status (*N* = 301)			< 0.001
EQ-VAS (range 0–100)	73	64	
Smoking (*N* = 300)			0.8
Current	18 (15%)	28 (15%)	
Former	62 (53%)	101 (56%)	
Never	38 (32%)	53 (29%)	
Alcohol (*N* = 300)			0.9
< 7/14	100 (85%)	153 (84%)	
> 7/14	18 (15%)	29 (16%)	

RCT: randomised controlled trial; SD: standard deviation; EQ-VAS: EuroQoL-Visual Analogue Scale.

* Ratio = number of participants divided by number of non-participants.

The mean age was significantly higher among non-participants compared to participants (*p* = 0.005), and significantly fewer women participated compared to men (*p* = 0.04). A significant difference between participants and non-participants was observed across hospitals.

No statistically significant difference in participation was observed across cancer subtypes. Although a slightly higher proportion of participants were diagnosed with oropharyngeal cancer (91% vs. 81%), and hypopharyngeal cancer was more frequent among non-participants (12% vs. 3%), these differences were not statistically significant. No differences were observed for laryngeal or nasopharyngeal cancers.

There were no significant differences between participants and non-participants concerning tumour stage (T1–T4, *p* = 0.2), nodal stage (N0–N3, *p* = 0.08), or overall clinical stage (Stage I–IV, *p* = 0.7).

Among respondents with oropharyngeal cancer (*n* = 254), Human papillomavirus status (measured by p16 immunohistochemistry) did not differ significantly between participants and non-participants. The proportion of p16-positive patients was 87% among participants and 81% among non-participants (*p* = 0.09).

Regarding health-related characteristics, there were no significant differences in comorbidity burden (*p* = 0.2), with similar proportions reporting no comorbid conditions or at least one. Similarly, no significant differences were observed in smoking status (*p* = 0.8) or alcohol consumption (*p* = 0.9).

**Figure 2:**
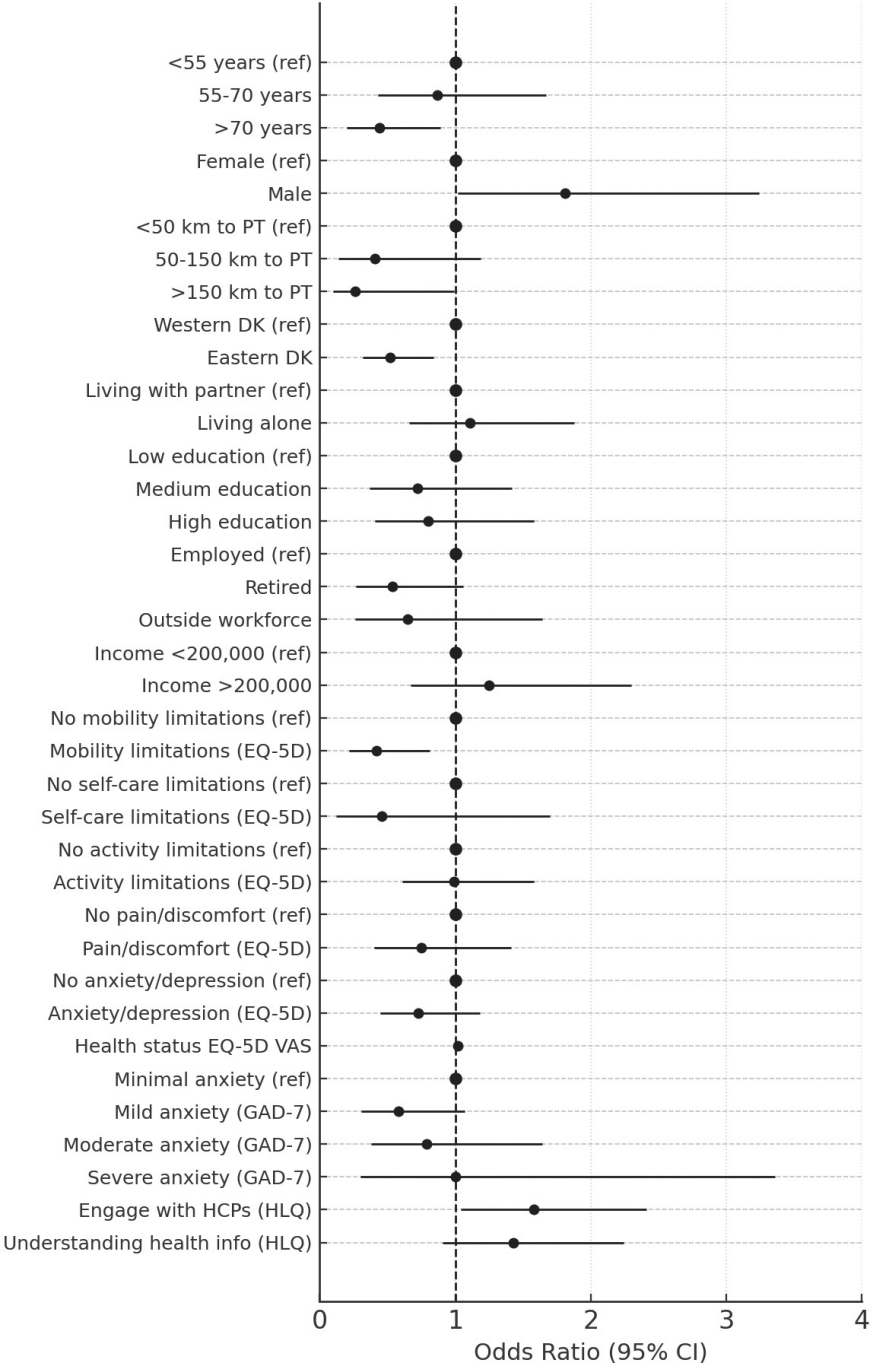
Adjusted odds ratios for participation in the DAHANCA 35 study, based on multivariable logistic regression models. DAHANCA: Danish Head and Neck Cancer Study Group; PT: proton therapy; VAS: visual analogue scale; EQ: EuroQoL; HLQ: Health Literacy Questionnaire; CI: confidence intervals.

Associations between exposure variables and participation in DAHANCA 35 are presented as crude and adjusted OR, with adjustments made for age and sex (male/female) (Table 3) (Figure 2). Higher OR indicate a greater likelihood of participation.

**Table 3:** Associations between exposure variables and participation in Danish Head and Neck Cancer Study Group (DAHANCA) 35.

Exposure variables	Univariable		Multivariable*	
	OR	95% CI	OR	95% CI
Age (*N* = 304)	0.96	0.94–0.99		
Age (*N* = 304)				
< 55	Ref			
55–70	0.87	0.43–1.67		
> 70	0.44	0.20–0.89		
Gender (*N* = 304)				
Female	Ref			
Male	1.81	1.02–3.24		
Km to the proton therapy (*N* = 233)	0.996	0.993–0.998	0.996	0.993–0.998
Km to proton therapy (*N* = 233)				
< 50 km	Ref		Ref	
50–150	0.33	0.12–0.93	0.41	0.14–1.19
> 150 km	0.23	0.08–0.61	0.26	0.10–0.99
Difference in km between RT modalities (photon/proton)				
0 km	Ref		Ref	
1–50 km	0.82	0.31–2.17	0.92	0.34–2.49
51–100 km	0.33	0.07–1.60	0.46	0.09–2.30
> 100 km	0.30	0.13–0.66	0.32	0.14–0.73
Part of country (*N* = 304)				
Western Denmark	Ref		Ref	
Eastern Denmark	0.54	0.34–0.86	0.52	0.32–0.84
Civil status (*N* = 300)				
Living with a partner	Ref		Ref	
Living alone	0.97	0.59–1.60	1.11	0.66–1.88
Education (*N* = 304)				
Low	Ref		Ref	
Medium	0.76	0.39–1.48	0.72	0.37–1.42
High	0.75	0.39–1.45	0.80	0.41–1.58
Employment (*N* = 303)				
Within workforce	Ref		Ref	
Retired (age)	0.42	0.25–0.70	0.54	0.27–1.06
Outside workforce	0.61	0.24–1.54	0.65	0.26–1.64
Income (*N* = 300)				
< 200,000 DKK	Ref		Ref	
> 200,000 DKK	1.67	0.95–2.94	1.25	0.67–2.30
EQ-5D Mobility (*N* = 301)				
No problems	Ref		Ref	
Any problems	0.36	0.19–0.69	0.42	0.22–0.81
EQ-5D Self-care (*N* = 301)				
No problems	Ref		Ref	
Any problems	0.45	0.12–1.66	0.46	0.12–1.70
EQ-5D Activities (*N* = 301)				
No problems	Ref		Ref	
Any problems	1.06	0.67–1.68	0.99	0.61–1.58
EQ-5D Pain (*N* = 301)				
No problems	Ref		Ref	
Any problems	0.76	0.41–1.39	0.75	0.40–1.41
EQ-5D Anxiety (*N* = 301)				
No problems	Ref		Ref	
Any problems	0.69	0.43–1.10	0.73	0.45–1.18
Health status (*N* = 301), VAS 0–100	1.02	1.01–1.04	1.02	1.01–1.03
Anxiety (*N* = 298), GAD-7 sum 0–21	0.96	0.91–1.01	0.97	0.92–1.02
Anxiety (*N* = 298)				
Minimal	Ref		Ref	
Mild	0.51	0.27–0.92	0.58	0.31–1.07
Moderate	0.72	0.35–1.50	0.79	0.38–1.64
Severe	0.88	0.27–2.89	1.00	0.30–3.36
HLQ scale 6: Actively engage with healthcare providers (*N* = 304) (sum 1–5)	1.75	1.16–2.65	1.58	1.04–2.41
HLQ scale 9: Understanding health information (*N* = 303) (sum 1–5)				

VAS: visual analogue scale; EQ: EuroQoL; HLQ: Health Literacy Questionnaire; CI: confidence intervals; GAD: Generalised Anxiety Disorder; OR: odds ratios.

* Adjusted for age and sex.

Age was significantly associated with participation when analysed as a continuous variable. Each 1-year increase in age was associated with a 4% decrease in the odds of participation (OR = 0.96, 95% CI: 0.94–0.99).

Individuals living more than 150 km from the PT centre had significantly lower odds of participating (OR = 0.26, 95% CI: 0.10–0.99), with each additional kilometre being associated with a 0.4% reduction in the odds of participation. Furthermore, a greater travel distance to the proton compared to the photon facility was associated with lower odds of participation. Patients with > 100 km additional distance had significantly lower odds of participation (OR = 0.32, 95% CI: 0.14–0.73).

The adjusted analysis found no statistically significant associations between socioeconomic variables of civil status, education or income and participation.

Regarding psychosocial and health-related factors, participants reporting mobility problems (EQ-5D dimension) had significantly lower odds of participation compared to those without mobility issues (OR = 0.42, 95% CI: 0.22–0.81). No significant associations were found for the remaining EQ-5D dimensions of self-care, usual activities, pain/discomfort, or anxiety/depression.

Higher self-rated health status was significantly associated with increased odds of participation, with a 2% increase with each one-point increase on the EQ-5D VAS scale (OR = 1.02, 95% CI: 1.01–1.03). The mean VAS score was also significantly higher among participants compared to non-participants (73 vs. 64, *p* < 0.001), indicating a nine-point difference.

Anxiety assessed with the GAD-7 was not significantly associated with participation, whether modelled as a continuous score or as categorical levels. Although a non-significant trend was observed for lower participation among individuals with mild to moderate anxiety compared to those with minimal symptoms, the association did not reach statistical significance.

Health literacy (HLQ Scale 6: actively engaging with healthcare providers) was significantly associated with participation (OR = 1.58, 95% CI: 1.04–2.41, *p* = 0.03), whereas no significant association was found for HLQ Scale 9 (understanding health information) in the adjusted model.

## Discussion

This nationwide cross-sectional survey investigated socioeconomic, geographical, and psychosocial factors associated with participation in the Danish DAHANCA 35 RCT, comparing proton and photon radiotherapy for pharyngeal and laryngeal cancer. The distribution of age, gender, tumour site, p16 status, and T and N stage appeared consistent with the broader Danish head and neck cancer population [35–38].

Female patients had lower odds of participation, consistent with previous findings showing gender disparities in clinical trial enrolment [10]. Older age was also associated with reduced participation. Prior research suggests this may partly reflect lower eligibility among older patients [15, 39]. Age and sex were included as covariates in the adjusted model.

Living in the eastern part of Denmark, furthest from the PT centre, and having a travel distance of more than 150 km were significantly associated with lower participation. This supports previous findings that geographical distance is a structural barrier to trial enrolment, particularly when the study treatment is delivered at a centralised facility. In this study, access to standard photon-based radiotherapy was similar across groups, with no significant difference in median travel distance. However, access to the PT facility differed markedly: participants lived significantly closer to the PT centre than non-participants. Furthermore, only patients with more than 100 km additional travel distance to PT centre, compared to their local photon clinic, had significantly lower odds of participation. This suggests that it is not the absolute travel burden alone, but the relative additional distance to the centralised treatment that functions as a structural barrier. These results highlight the importance of considering patient-perceived ‘extra burden’ when designing multicentre trials involving centralised study treatments. Distance adds complexity when conducting RCTs in such settings [11–13, 40]. For head and neck cancer, PT requires treatment 5 days a week over 6 weeks, and longer travel or the need for accommodation may increase the treatment burden. Prior qualitative work has shown that being away from home can contribute to reluctance to enrol [21].

Mobility limitations and lower self-rated health were associated with lower rate of participation. This aligns with studies showing that patients with reduced physical function are less likely to participate in trials [10, 16, 39]. In the current study, the mean EQ-VAS score was 73 among participants and 64 among non-participants – a 9-point difference. This exceeds the minimally important difference (MID) established in cancer populations and may therefore be clinically meaningful [41].

Health literacy (ability to actively engage with healthcare providers) remained significant in the adjusted model. Limited health literacy has previously been identified as a barrier due to challenges in understanding trial information and communicating with clinicians [16, 18, 42]. Improving health literacy responsiveness, defined as the ability of healthcare systems and professionals to recognise and accommodate patients’ varying health literacy levels, may help ensure that patients with lower health literacy are better supported in understanding and engaging with trials [43].

Although socioeconomic variables such as income, education, and marital status were not statistically significant, these factors have been associated with trial participation in prior studies [11, 14, 17]. The lack of statistical significance may reflect limited power. However, it may also reflect contextual strengths of the Danish healthcare system, including tax-funded universal access, trial recruitment efforts in the radiotherapy clinics, and efficient coordination between regional radiotherapy clinics and the PT centre. These structural and systemic features may mitigate the influence of socioeconomic factors. The findings may also illustrate that population-level socioeconomic patterns do not necessarily translate into meaningful clinical predictors at the individual level. While structural inequalities persist, indicators such as income or education may not reliably guide clinical efforts to support participation.

The 41 patients who reported not being informed about the DAHANCA 35 trial were distributed across all six centres, ranging from 7% to 22% of respondents per site. This centre variation may reflect local differences in trial communication practices. While this group was retained in the main analysis, a sensitivity analysis excluding these patients was conducted. The results remained consistent, with no changes in the pattern of significant associations.

Some scales showed response patterns suggestive of ceiling or floor effects. HLQ Scale 6 had a high proportion of maximum scores (23%), indicating a potential ceiling effect. On GAD-7, 23% scored zero, suggesting a floor effect. While these patterns may partly reflect limited scale sensitivity, they could also result from selection bias, with respondents potentially less anxious and more resourceful than non-respondents. Thus, limited variability may reflect sample characteristics rather than instrument limitations, potentially affecting generalisability.

There was a risk of selection bias at two levels: questionnaire distribution and patient response. Regarding distribution, Health care professionals (HCPs) may have found it easier to approach patients they perceived as more capable of completing the survey or those already enrolled in DAHANCA 35, who were familiar with the RCT and PT. This is supported by the variation in participant-to-non-participant ratios across centres (Table 2), suggesting that in some clinics, DAHANCA 35 participants were more frequently invited to participate in the survey. As a result, only a subset of eligible patients received the questionnaire, potentially introducing selection bias if certain groups were systematically underrepresented. Survey response rates also varied across centres, likely reflecting local differences in how the questionnaire was introduced and whether HCPs followed up to encourage its completion. In terms of response, patients’ participation in DAHANCA 35, willingness to engage with questionnaires, and personal circumstances may have influenced completion, potentially contributing to variation in response patterns. Although representativeness remains uncertain, the data provide valuable insight into a group that is typically hard to reach in research.

The study’s strengths included using validated patient-reported outcome measures and linkage with high-quality clinical data from the national DAHANCA database [26]. These measures enabled the exploration of psychosocial characteristics, including health literacy, anxiety, and perceived health, which are unavailable in clinical registries. In addition, geographical disparity in access to the proton centre was assessed with relatively high precision using patients’ postal codes, providing a clear distance gradient as a structural barrier.

Limitations included a limited number of respondents, which reduced statistical power. Although the sample size (*N* = 363) exceeded the pre-specified target of 330, 59 individuals were excluded prior to analysis, resulting in a final sample of 304.

The clinical implications of this study relate to identifying factors that may limit trial enrolment, particularly in centralised treatment settings. The findings provide important knowledge about who may be excluded and emphasise the need to design trials that minimise structural and psychosocial barriers. While such barriers cannot be fully eliminated, they can be addressed through patient-centred communication, support, and logistical assistance.

## Conclusion

This nationwide survey identified factors associated with participation in DAHANCA 35. Female gender, older age, greater geographical distance to the treatment centre, mobility limitations, lower self-rated health status, and lower ability to actively engage with healthcare providers were all associated with reduced participation. These findings highlight the need for interventions that promote equitable access to PT trials. This includes health literacy-responsive approaches that enhance trial communication and decision support, trial designs that facilitate broad inclusion, and logistical solutions for patients undergoing long-term treatment away from home, including transportation and accommodation arrangements.

## Data Availability

The data underlying this study are stored in REDCap at Aarhus University. Data may be made available from the first author upon reasonable request and with appropriate institutional approvals.
